# Active Minds, Healthy Bodies: Investigating Physical Activity, Barriers, and Health Outcomes in Saudi Adolescent Females

**DOI:** 10.3390/children12070846

**Published:** 2025-06-27

**Authors:** Wafaa Saleh, Ghada Alturif

**Affiliations:** 1 Transport Research Institute, School of Computing, Engineering and the Built Environment, Edinburgh Napier University, Edinburgh EH10 5DT, UK; 2 Department of Social Work, College of Humanities and Social Sciences, Princess Nourah Bint Abdulrahman University, P.O. Box 84428, Riyadh 11671, Saudi Arabia; gaaltarif@pnu.edu.sa

**Keywords:** adolescents, physical activity, Saudi Arabia, PAQ-A, SEM, Ordinal Logistic Model (OLM), Self-Rated Health (SRH), environmental barriers

## Abstract

**Background:** Physical inactivity among adolescents, particularly females in conservative societies like Saudi Arabia, poses significant risks to both physical and psychological health. Cultural norms, environmental constraints, and limited access to gender-appropriate sports facilities further exacerbate the problem. Understanding these factors is essential for developing effective, context-sensitive interventions. **Aim:** This study aimed to examine the relationship between daily physical activity behaviours and health outcomes, both physical and psychological, among adolescent females in Saudi Arabia. This paper also explores the impact of socio-environmental variables such as income, household size, and urban/rural residence on activity levels and wellbeing. **Methods:** A cross-sectional survey of 636 adolescent females aged 13–21 was conducted using validated instruments. The Physical Activity Questionnaire for Adolescents (PAQ-A) measured physical activity levels, and the Self-Rated Health (SRH) assessed psychological wellbeing and health-related quality of life. The analysis employed an Ordinal Logistic Model (OLM) guided by the Social Ecological Model (SEM) to assess predictors of physical activity participation. **Results:** The OLM results indicated that higher income levels, smaller household size, urban residence, and older age categories were significantly associated with higher levels of physical activity (*p* < 0.05). Participants with higher PAQ-A scores reported better psychological health and quality of life. The model showed a good fit, with a pseudo-R^2^ of 0.36. Cultural and environmental barriers, particularly in rural areas, were identified as significant deterrents to participation. **Conclusions:** The findings highlight the urgent need for culturally adapted, gender-sensitive health promotion programs that target both individual and structural barriers to physical activity among adolescent girls in Saudi Arabia. Enhanced societal support and accessible sports infrastructure could significantly improve health outcomes and quality of life.

## 1. Introduction

Regular physical activity is a critical determinant of physical, psychological, and social wellbeing among children and adolescents. It supports healthy growth and development, reduces the risk of non-communicable diseases, and enhances mental health and quality of life [1,2,3,4,5]. However, global trends show a marked decline in activity levels among youth, with adolescent girls particularly at risk of physical inactivity due to intersecting social, environmental, and cultural barriers [6].

In Saudi Arabia, these challenges are exacerbated by unique sociocultural dynamics, including restrictive gender norms, limited access to female-friendly sports facilities, and the inconsistent implementation of physical education programs for girls [7,8]. Although national initiatives such as Saudi Vision 2030 are promoting inclusive and health-oriented lifestyles, adolescent females still face substantial structural and cultural obstacles to regular physical activity [9]. These realities highlight the urgent need for context-specific research to understand how social support, environmental access, and cultural attitudes intersect to influence the physical and psychological wellbeing of young Saudi females.

While the international literature consistently links physical activity with improved health outcomes, including better mood, reduced anxiety, higher self-esteem, and improved cardiovascular fitness [10,11,12,13,14,15,16,17,18], such relationships remain underexplored in the Saudi context—particularly among adolescent girls. Evidence from other regions shows that sports participation supports not only physical and mental health, but also social skills, discipline, and a sense of accomplishment [13,14,15,16,17,18]. Nevertheless, Saudi-specific research remains limited in scope, often lacking in-depth exploration of the sociocultural factors that inhibit or facilitate adolescent girls’ participation in physical activity.

The gender gap in physical activity is particularly pronounced in Saudi Arabia, where cultural expectations around femininity and modesty restrict opportunities for girls to engage in public or organized sports [7,19]. Structural barriers such as the scarcity of girls-only facilities, safety concerns, and lack of supportive school programs further reduce participation levels. Guthold et al. [6] reported that more than 80% of adolescents worldwide fail to meet WHO recommendations for daily activity, with girls consistently reporting lower engagement than boys. These disparities are compounded by familial and community attitudes that may discourage girls from engaging in physical activity, leading to reduced psychological wellbeing and lower perceived quality of life [19,20].

Given these multifaceted influences, researchers increasingly recommend mixed-methods approaches that combine the statistical rigor of quantitative tools with the contextual depth of qualitative inquiry [21,22]. This study follows that approach, employing both systematic surveys and semi-structured interviews to capture not only the frequency of activity but also the lived experiences, perceptions, and cultural narratives that shape participation. The Social Ecological Model (SEM) [23] provides a guiding framework, organizing the inquiry across multiple levels: individual (e.g., motivation and perceived health), interpersonal (e.g., family support), environmental (e.g., access to facilities), and societal (e.g., gender norms and policy context).

The SEM is particularly suited to the Saudi context, where personal choices are often embedded within strong familial, educational, and community structures. Applying the SEM allows for a nuanced analysis of how these factors interact to shape behaviour and offers insight into potential leverage points for culturally sensitive interventions. For example, at the individual level, Self-Rated Health (SRH)—a validated and widely used single-item measure of perceived health—has proven predictive of both physical activity engagement and long-term health outcomes [24,25,26,27,28,29]. At the interpersonal and community levels, variables such as parental education, family encouragement, and urban residence can either support or constrain girls’ activity levels.

To quantify these relationships, this study used the Physical Activity Questionnaire for Adolescents (PAQ-A) [10], a validated self-report instrument widely used across cultural contexts, including the Middle East. It categorizes respondents into low, moderate, and high activity levels over a 7-day period. Using this instrument, an *Ordinal* Logistic Model (OLM) was applied to assess how SEM-informed variables predict activity frequency. Complementing this, qualitative interviews explored adolescents’ perceptions of barriers and supports, adding depth to the quantitative findings and enhancing cultural relevance.

Building on the national momentum of Vision 2030—which explicitly emphasizes women’s empowerment and increased physical activity—this study aims to contribute empirically grounded recommendations to policymakers, educators, and public health leaders seeking to enhance adolescent girls’ wellbeing through active living.

### Research Aim and Questions

The overall aim of this study is to investigate the key factors influencing physical activity participation and wellbeing among adolescent females in Saudi Arabia. Guided by the Social Ecological Model and using the validated PAQ-A instrument, the study explores how individual, social, cultural, and environmental factors relate to activity behaviours and perceived quality of life. The study addresses the following three research questions:

1. What individual, social, and environmental factors significantly influence physical activity participation among adolescent females in Saudi Arabia?
2. How do cultural attitudes, family support, and access to facilities relate to the psychological wellbeing and quality of life of physically active and inactive adolescent females?
3. How can the findings inform culturally sensitive interventions to promote physical activity and enhance quality of life among young females in Saudi Arabia?

By addressing these questions, this research aims to generate evidence-based insights to support culturally tailored strategies that promote physical activity and health equity for adolescent girls across the Kingdom.

## 2. Methodology

### 2.1. Study Area

This study targeted adolescent females aged 13–21 in the Kingdom of Saudi Arabia, with the aim of capturing diverse physical activity behaviours across varying sociocultural contexts. A total of 636 participants were recruited from both urban and semi-urban areas across four major regions: Riyadh, Jeddah, Dammam, and their surrounding towns. These locations were strategically selected to reflect different levels of access to physical activity infrastructure, cultural attitudes towards female sports participation, and socioeconomic diversity. The selection criteria ensured the inclusion of students from public and private schools, community centres, and health programs, enabling a broad representation of educational, economic, and geographic backgrounds. The age range was chosen to encompass middle to late adolescence and early adulthood, a period critical for identity formation, lifestyle development, and health behaviour establishment.

Participants were selected using stratified purposive sampling to ensure proportional representation from each selected region, taking into account school type, area classification (urban vs. semi-urban), and socioeconomic indicators.

### 2.2. Study Design

This study adopted a mixed-methods design to thoroughly explore physical activity behaviours, perceived social and cultural supports and barriers, and related physical and psychological health outcomes among adolescent females in Saudi Arabia. The design is guided by the Social Ecological Model (SEM), which provides a framework for understanding how individual behaviour is influenced by a constellation of factors across interpersonal, organizational, community, and policy levels [23]. Rooted in Bronfenbrenner’s ecological systems theory [30] and adapted for public health [23], the SEM enables the study to capture the complex interplay between personal attributes (e.g., age and motivation), social relationships (e.g., family and peer support), environmental conditions (e.g., access to recreational facilities and school programs), and broader cultural norms. These layers were examined across five domains: individual, interpersonal, organizational, community, and policy.

By applying the SEM, this study identifies multiple levels at which interventions can be implemented to support adolescent girls’ engagement in physical activity. This comprehensive framework shaped the design of the survey instrument and guided the analysis of how barriers and facilitators operate across various settings. The Physical Activity Questionnaire for Adolescents (PAQ-A) was used to assess physical activity levels, capturing self-reported behaviours over a seven-day period, while the Self-Rated Health (SRH) instrument provided a measure of perceived physical and psychological wellbeing. This integration allowed for deeper insight into how contextual and personal determinants intersect to influence physical activity in a culturally specific setting.

Importantly, SRH contributed to identifying at-risk individuals and aligned with the SEM by linking personal health perception with broader ecological influences [24,25,26,27,28,29]. Its cultural adaptability and simplicity enhance its value as a public health tool, especially where standardized instruments like the Pediatric Quality of Life Inventory (PedsQL) are not feasible [31].

To quantitatively assess the relationship between physical activity and its predictors, an Ordinal Logistic Model (OLM) was employed. The dependent variable captured three ordered levels of physical activity: Level 1 (physically inactive or rarely active), Level 2 (occasionally active), and Level 3 (regularly active). Independent variables were derived from the conceptual framework and included age group, cultural barriers (low, medium, or high), family support (present or absent), access to facilities (adequate or inadequate), safety concerns (high or low), socioeconomic status (SES), parental education level, psychological health status, and urban vs. rural residence. SRH was modelled as an ordinal predictor to reflect respondents’ perceived health and its role in shaping behaviour.

The OLM estimated the log odds of being at or below each physical activity level, allowing the study to identify how various multilevel factors influence participation. Model calibration was based on the log-likelihood, Akaike Information Criterion (AIC), and pseudo R^2^ values to evaluate the model fit and predictive strength [32]. This modelling approach supports a nuanced, context-specific understanding of adolescent females’ physical activity behaviours and informs culturally responsive strategies for intervention in Saudi Arabia.

The model is specified as follows:

Logit [P(Y ≤ d)] = αd − ∑βnXn

where

Y is the ordinal dependent variable representing the level of physical activity participation (Level 1, 2, or 3);

d indicates the cutoff points between these ordered levels;

Xn are the independent variables (predictors);

βn are the estimated coefficients for each predictor;

αd are the intercepts (threshold parameters) for each cumulative logit.

### 2.3. Data Collection Procedures

The study population consisted of female adolescents aged 13 to 21 years enrolled in public and private colleges located in urban and suburban areas of Saudi Arabia. To ensure diverse representation across institutional types and socioeconomic backgrounds, a stratified random sampling strategy was employed. The target sample size for the quantitative component was approximately 636 participants, providing adequate statistical power for the analyses. In addition, a purposive subsample of 30 participants was invited to participate in qualitative, in-depth interviews to gain richer contextual insights.

Data collection was conducted using a structured, self-administered online questionnaire designed to investigate physical activity behaviours, psychosocial determinants, and health-related quality of life among adolescent females. The questionnaire incorporated validated instruments, most notably the Physical Activity Questionnaire for Adolescents (PAQ-A), which assesses the frequency and intensity of physical activity over a seven-day period. The instrument was structured into four main sections:

1. Demographic Information—including participants’ age, type of educational institution, urban or rural residence, socioeconomic status (SES), and parental education level.
2. Physical Activity Patterns—assessing the types of physical activity undertaken, daily and weekly frequency, and indicators of sedentary behaviour.
3. Social and Environmental Influences—measuring perceived support from family and school, access to recreational or sports facilities, and perceptions of neighbourhood safety.
4. Cultural and Psychological Factors—evaluating attitudes toward female participation in physical activity, mental wellbeing, and cultural barriers identified through the prior literature and expert consultations.

For analytical purposes, data were categorized into key variables that informed the model estimation:

- Age Group: 13–15, 16–18, and 19–21 years.
- Cultural Barriers: High, medium, or low (based on self-reported perceptions of sociocultural limitations).
- Family Support: Yes or no (reflecting the presence of encouragement or logistical support from family members).
- Access to Facilities: Adequate or inadequate (based on participants’ assessment of facility availability and suitability).
- Safety Concerns: High or low (based on perceived safety of the local environment for physical activity).
- Socioeconomic Status (SES): High, medium, or low (derived from reported household income or related indicators).
- Parental Education Level: University, secondary, or other (based on the highest level attained by either parent).
- Psychological Health Status: Good, moderate, or poor (measured through self-reported mental wellbeing).
- Residence Type: Urban or rural (defined by geographic classification).

The survey was administered in Arabic to ensure cultural and linguistic relevance. Trained female researchers facilitated data collection and conducted qualitative interviews where applicable, ensuring a respectful and culturally sensitive approach. Interviews were audio-recorded with participants’ consent and transcribed verbatim to support detailed thematic analysis.

### 2.4. Ethical Considerations

The study protocol was approved by the Institutional Review Board at Princess Nourah bint Abdulrahman University in Saudi Arabia. Informed consent and assent procedures ensured participants’ understanding and voluntary participation. Ethical approval was obtained through Princess Nourah bint Abdulrahman University, and parental consent was secured for all participants under the age of 18. Confidentiality was maintained by anonymizing data, and participants were informed of their right to withdraw at any time without penalty.

## 3. The Results

The data presented in Table 1, Table 2 and Table 3 provide a comprehensive overview of the physical activity patterns, barriers, and health outcomes among adolescent females in Saudi Arabia, shedding light on critical factors influencing their engagement in physical activities. Table 1 shows the physical activity frequency among adolescent females. Table 2 shows the prevalence of barriers by physical activity frequency. Table 3 presents the psychological and physical health outcomes reported. Table 4 presents the coefficient estimates of independent variables, odds ratios, and goodness-of-fit statistics of the Ordered Logit Model. These results are discussed in the next section (Section 5).

## 4. Analysis of the Results

### 4.1. General Analysis

The study surveyed 636 adolescent females regarding their physical activity frequency and perceived barriers to participation. The data presented in Table 1, Table 2 and Table 3 provide a comprehensive overview of the physical activity patterns, barriers, and health outcomes among adolescent females in Saudi Arabia, shedding light on critical factors influencing their engagement in physical activities. Table 1 shows the distribution of physical activity frequency and reveals that only 15% of adolescent females meet the recommended daily exercise threshold, while the majority (60%) engage in physical activity sporadically or rarely. This low adherence aligns with global trends of insufficient physical activity among adolescent girls, particularly in conservative cultural contexts, and underscores the urgent need for targeted interventions to promote regular activity. Table 2 shows that cultural restrictions emerge as the most pervasive barrier across all activity levels, increasing from 70% among the most active group to 90% among the least active. This finding highlights the substantial influence of sociocultural norms in limiting physical activity opportunities for females. Additionally, lack of family support significantly contributes to inactivity, suggesting that family attitudes and encouragement are pivotal in shaping adolescent behaviour. Environmental barriers such as inadequate facilities and safety concerns also exhibit a clear gradient, with higher prevalence reported among less active participants. These results indicate that multifaceted barriers, spanning cultural, social, and environmental domains, interact to discourage consistent physical activity.

Finally, Table 3 indicates that positive health outcomes such as improved mood (60%) and reduced stress (55%) were reported by a substantial portion of participants, reinforcing the well-established psychological benefits of regular physical activity. Increased physical fitness was noted by half of the respondents, illustrating tangible physical benefits. However, the presence of fatigue (30%) and pain (20%) among some participants may reflect challenges related to inadequate preparation, overexertion, or lack of proper facilities, suggesting the importance of safe and guided physical activity programs. These findings highlight a strong inverse relationship between physical activity frequency and the perceived intensity of sociocultural and environmental barriers. Participants with lower activity levels consistently reported higher prevalence of all barriers, suggesting that these factors play a significant role in limiting physical activity among adolescent females in Saudi Arabia. Together, these findings emphasize the complex interplay between behavioural patterns, contextual barriers, and health outcomes. Interventions aiming to increase physical activity among Saudi adolescent females must address cultural sensitivities, enhance family and community support, and improve infrastructure and safety measures. Moreover, promoting awareness of the mental and physical health benefits could motivate greater participation and adherence.

The adoption of the Ordinal Logistic Model (OLM) in this study is well justified by the nature of the dependent variable, physical activity frequency, which was categorized into ordered levels (i.e., never, occasionally, regularly, daily). Unlike continuous or binary outcomes, this ordinal structure necessitates a statistical approach that accounts for the inherent order without assuming equal spacing between categories. The OLM provides cumulative odds ratios, which offer intuitive and interpretable results about how changes in each independent variable affect the odds of an adolescent engaging in more frequent physical activity. This is particularly valuable in public health research, where understanding the direction and magnitude of influence across ordinal levels informs targeted intervention design. In this study, the OLM allowed for the calibration of a multifactorial model that aligned with the Social Ecological Model (SEM), capturing individual, interpersonal, and environmental determinants simultaneously. The model’s goodness-of-fit statistics indicated satisfactory model performance, and key predictors such as cultural barriers, family support, and psychological wellbeing were found to be significant, supporting the validity and utility of the OLM in this context.

The Ordinal Logistic Model (OLM) was calibrated to examine the levels of physical activity participation among adolescent females in Saudi Arabia and their association with cultural, social, and environmental factors. Table 4 provides the coefficient estimates of the independent variables, odds ratios, and goodness-of-fit statistics of the Ordered Logit Model.

### 4.2. General Interpretation of OLM Results

The results of the Ordinal Logistic Model (OLM) revealed several statistically significant predictors of physical activity frequency among adolescent females, providing strong support for the theoretical application of the Social Ecological Model (SEM). Notably, Self-Rated Health (SRH) emerged as the strongest individual-level predictor. With a coefficient of 0.925 and a *p*-value of <0.001, each one-level increase in SRH (e.g., from “Fair” to “Good”) more than doubled the odds of reporting more frequent physical activity (OR = 2.52). This underscores the critical role of perceived health in motivating physical engagement. At the interpersonal level, family support was also highly significant (β = 0.841, *p* < 0.001, OR = 2.32), indicating that adolescents who received encouragement from family were over twice as likely to be more physically active. Environmental factors also had strong effects. Access to adequate facilities significantly increased the odds of higher activity (β = 0.710, *p* < 0.001, OR = 2.03), while high cultural barriers (β = −0.785, *p* < 0.001, OR = 0.46) and high safety concerns (β = −0.552, *p* = 0.007, OR = 0.58) were significant inhibitors. These findings highlight how physical and sociocultural environments can either facilitate or restrict physical activity among adolescent females, particularly in conservative settings. Additionally, adolescents from high socioeconomic backgrounds (β = 0.630, *p* = 0.003, OR = 1.88) and those whose parents had a university education (β = 0.527, *p* = 0.004, OR = 1.69) were significantly more likely to engage in frequent activity, indicating that social capital and educational attainment play important roles. Good psychological health was also associated with higher activity (β = 0.676, *p* < 0.001, OR = 1.97), further reinforcing the interplay between mental wellbeing and physical behaviour.

Among demographic controls, younger adolescents aged 13–15 were significantly more active (β = 0.482, *p* = 0.021, OR = 1.62) than those aged 19–21, suggesting that physical activity declines with age. Living in urban areas was also positively associated with activity frequency (β = 0.402, *p* = 0.026, OR = 1.50), likely reflecting better access and fewer cultural restrictions. Other variables, such as medium SES, moderate psychological health, and secondary parental education, were not statistically significant, although their directional effects were consistent with theoretical expectations. Overall, the model demonstrated a good fit (Nagelkerke R^2^ = 0.36), and the significance of predictors across multiple SEM levels supports the need for integrated, multilevel strategies to promote physical activity among adolescent girls.

### 4.3. Specific Interpretation of OLM Results

The OLM shows that a one-unit increase in the “age group (13–15)” variable results in a 0.482 increase in the log odds of being in a higher category of physical activity (e.g., occasionally active or regularly active), holding all other variables constant. The corresponding odds ratio is 1.62, indicating that adolescent females aged 13–15 are 62% more likely to be more physically active than those aged 19–21 (reference group). This finding may reflect the stronger integration of physical activity in younger adolescents’ routines or school environments. A one-unit increase in reported high cultural barriers corresponds to a 0.785 decrease in the log odds of being in a higher activity level. The odds ratio is 0.46, meaning that participants facing high cultural barriers are 54% less likely to be active compared to those reporting low barriers. This underscores the restrictive impact of societal norms and expectations on young females’ engagement in physical activity in conservative environments.

Participants reporting family support have a 0.841 increase in the log odds of being more physically active. With an odds ratio of 2.32, they are more than twice as likely to engage in physical activity compared to those without family support. This emphasizes the critical role of family encouragement and permission, especially in contexts where external social support may be limited. Having adequate access to facilities increases the log odds by 0.710, with an odds ratio of 2.03. This suggests that participants with adequate access are twice as likely to be more active, highlighting the importance of investment in female-friendly sports infrastructure. A high level of safety concern leads to a 0.552 decrease in log odds of being more active, with an odds ratio of 0.58. Adolescents concerned about safety are 42% less likely to participate in physical activity. This could reflect concerns about personal security, transportation, or privacy in facilities, which are especially relevant in the Saudi context. Those from high-income families show a 0.630 increase in log odds, with an odds ratio of 1.88. They are 88% more likely to participate in higher levels of physical activity. This may be due to greater access to private facilities, transportation, and paid programs.

Higher parental education (university level) increases the log odds of activity by 0.527, corresponding to an odds ratio of 1.69. This suggests that educated parents may be more aware of the health benefits of physical activity and more likely to encourage their daughters. A good level of psychological health corresponds to a 0.676 increase in log odds, with an odds ratio of 1.97. This means adolescents reporting good mental health are nearly twice as likely to be active, highlighting the bidirectional link between mental wellbeing and physical activity. Urban dwellers are 1.5 times more likely to participate in physical activity (β = 0.402, OR = 1.50). This is likely due to better availability of facilities, more social acceptance, and higher exposure to health campaigns in cities compared to rural areas. The model results show that the strongest positive predictors are family support, access to facilities, and psychological health. The greatest barriers are high cultural restrictions and safety concerns. Therefore, interventions should focus on improving access, addressing safety and cultural norms, and enhancing psychological support to promote greater participation in physical activity among adolescent females.

Estimating psychological health parameters in adolescent populations requires careful selection of reliable and validated tools that are age-appropriate and culturally sensitive. In the context of this study, psychological health was assessed through dimensions such as emotional wellbeing, self-esteem, and perceived stress, which are critical indicators of mental health in adolescence. To ensure robust estimation, responses were analysed using psychometric techniques (in this case, factor analysis and reliability testing) to confirm internal consistency (typically with Cronbach’s alpha > 0.70). Data were then integrated into multivariate analyses (which was the Ordinal Logistic Model) to examine how psychological health outcomes correlate with physical activity levels, sociodemographic variables, and environmental factors.

Additionally, attention was given to contextual influences, such as family support and college environments, which are particularly relevant in conservative societies like Saudi Arabia. This multi-layered approach ensures a more nuanced understanding of psychological health and its interaction with physical activity and societal support structures, strengthening the credibility and relevance of the findings. The urban/rural residence coefficient in the model captures the influence of geographic context on adolescent physical activity and related health outcomes. Its estimation is crucial because the living environment often dictates access to recreational facilities, school sports programs, transportation, and community safety, factors that significantly shape physical activity behaviours, especially among young females. In this study, urban and rural residence was treated as a categorical independent variable within the Ordinal Logistic Model (OLM). The coefficient reflects the odds of being in a higher physical activity category (e.g., active, club member, or event attendee) based on residence status. A positive and statistically significant coefficient for urban residence would suggest that adolescents in urban areas are more likely to be active, potentially due to greater infrastructure and social support for sports. Conversely, a negative or non-significant coefficient may highlight persistent barriers in urban environments, such as safety concerns or restrictive social norms, or the possible presence of informal activity opportunities in rural areas.

## 5. Discussions, Conclusions, and Limitations

This study examined the individual, social, and environmental determinants of physical activity among adolescent females in Saudi Arabia using a mixed-methods approach guided by the Social Ecological Model (SEM). The findings address the three research questions by identifying key predictors of physical activity (RQ1), exploring how cultural and psychosocial factors relate to wellbeing (RQ2), and outlining implications for culturally appropriate interventions (RQ3).

RQ1: Determinants of Physical Activity Participation

The results of the Ordinal Logistic Model (OLM) revealed that multiple factors across all SEM levels significantly influence physical activity participation. At the intrapersonal level, Self-Rated Health (SRH) emerged as the most influential predictor (β = 0.925, *p* < 0.001, OR = 2.52), indicating that adolescents who perceived themselves as healthier were more likely to be physically active. Psychological wellbeing (β = 0.676, *p* < 0.001, OR = 1.97) and a younger age (13–15 years) (β = 0.482, *p* = 0.021, OR = 1.62) were also significant, highlighting the interconnected nature of mental health and age in shaping activity patterns. These findings confirm previous evidence suggesting that physical activity tends to decline with age during adolescence [33,34].

At the interpersonal level, family support was a critical enabler (β = 0.841, *p* < 0.001, OR = 2.32). Adolescents with familial encouragement were over twice as likely to engage in regular physical activity. This reinforces the importance of social networks, particularly in cultural contexts where gender norms may restrict female mobility or public visibility [5,23,31,34].

At the environmental and societal levels, access to facilities (β = 0.710, *p* < 0.001, OR = 2.03) had a strong positive effect. In contrast, high cultural barriers (β = −0.785, *p* < 0.001, OR = 0.46) and high safety concerns (β = −0.552, p = 0.007, OR = 0.58) were negative predictors. These findings suggest that even when individual motivation exists, structural and societal constraints may prevent regular participation in physical activity.

Additional significant predictors included high SES (β = 0.630, p = 0.003, OR = 1.88), university-educated parents (β = 0.527, p = 0.004, OR = 1.69), and urban residence (β = 0.402, p = 0.026, OR = 1.50), indicating that socioeconomic advantage and geographic location can enable or constrain access to resources that facilitate physical activity.

RQ2: Cultural, Family, and Facility Access in Relation to Wellbeing

The strong associations between SRH, mental health, and physical activity levels demonstrate that psychological wellbeing is both a determinant and an outcome of physical activity. Participants who faced fewer cultural and safety constraints were more likely to report both higher activity levels and better psychological health. These findings highlight the burden of sociocultural restrictions and inadequate infrastructure on adolescent females’ mental and emotional wellbeing.

Family support not only promoted physical engagement but also correlated with improved self-perceptions of health, underscoring the role of emotionally and logistically supportive environments. Similarly, accessible and safe recreational spaces appear to mitigate psychological distress by enabling physical expression and peer interaction. These results support the growing consensus that mental wellbeing and physical health are mutually reinforcing and must be addressed together in youth-focused public health efforts [31,32,33,34].

RQ3: Implications for Culturally Sensitive Interventions

The findings offer clear guidance for designing multilevel, culturally sensitive interventions. At the individual level, strategies should promote mental wellbeing alongside physical activity by integrating health education and psychological support into school curricula. At the family level, programs should engage parents, particularly mothers, as advocates and facilitators of physical activity.

At the community level, investments in safe, female-friendly recreational spaces are critical, especially in suburban and rural areas. At the policy level, addressing gender-based restrictions and reforming school and municipal planning policies to include inclusive sports programming can create systemic support for adolescent girls.

Given the cultural sensitivities and structural limitations identified, effective interventions must go beyond education and awareness campaigns. They should be embedded in culturally appropriate delivery models, such as through schools, mosques, or family-oriented community centres, and emphasize safe, supportive, and gender-sensitive environments.

Several limitations should be acknowledged. First, the cross-sectional design limits causal inference. Longitudinal studies are necessary to understand how physical activity patterns and barriers evolve over time. Second, the study relied on self-reported data via the PAQ-A, which may be subject to recall and social desirability bias, particularly in a context where female physical inactivity is socially discouraged. The inclusion of objective measures like wearable trackers in future research would enhance data accuracy.

Third, although the sample was stratified by institution type and region, it may not fully capture the experiences of adolescents in remote rural areas, where barriers may differ significantly. Fourth, the qualitative subsample of 30 participants, while valuable for contextual insights, limits the generalizability of those narratives.

In conclusion, this study provides a comprehensive, evidence-based understanding of the multifactorial influences on physical activity among adolescent females in Saudi Arabia. By addressing the interplay of individual, social, and environmental factors, the findings affirm the relevance of the Social Ecological Model in culturally specific contexts. Public health efforts must target multiple levels, including health education, family engagement, facility access, and cultural transformation, to effectively promote adolescent female physical activity and overall wellbeing.

Future research should expand to underrepresented regions, include male perspectives, and evaluate the role of schools and health policies as systemic facilitators. The development and testing of culturally grounded interventions will be crucial to shifting societal norms and improving long-term health outcomes for adolescent girls across Saudi Arabia.

## Figures and Tables

**Table 1:** Physical activity frequency among adolescent females.

Physical Activity Frequency	Percentage (%)
Daily (≥5 days/week)	15
Several times per week (3–4 days/week)	25
Occasionally (1–2 days/week)	35
Rarely/Never	25

**Table 2:** Prevalence of barriers by physical activity frequency.

Barrier	Daily (%)	Several Times/Week (%)	Occasionally (%)	Rarely/Never (%)
Cultural Restrictions	70	80	85	90
Lack of Family Support	65	72	78	85
Inadequate Facilities	55	62	70	80
Safety Concerns	40	45	55	70

**Table 3:** Psychological and physical health outcomes reported.

Health Outcome	Percentage Reporting (%)
Improved Mood	60
Reduced Stress	55
Increased Physical Fitness	50
Experience of Fatigue	30
Experience of Pain	20

**Table 4:** Coefficient estimates of independent variables, odds ratios, and goodness-of-fit statistics of the Ordered Logit Model.

Predictor	Coefficient (β)	Std. Error	z-Value	*p*-Value	Odds Ratio (Exp(β))
Intercept (Level 1 vs. 2 and 3)	−1.812	0.410	−4.42	<0.001	—
Intercept (Level 1 and 2 vs. 3)	0.935	0.390	2.40	0.016	—
Age Group (13–15)	0.482	0.210	2.30	0.021	1.62
Age Group (16–18)	0.235	0.190	1.24	0.213	1.27
Cultural Barriers (High)	−0.785	0.225	−3.49	<0.001	0.46
Cultural Barriers (Medium)	−0.312	0.200	−1.56	0.119	0.73
Family Support (Yes)	0.841	0.195	4.31	<0.001	2.32
Access to Facilities (Adequate)	0.710	0.180	3.94	<0.001	2.03
Self-Rated Health	0.925	0.128	7.23	<0.001	2.521
Safety Concerns (High)	−0.552	0.205	−2.69	0.007	0.58
Socioeconomic Status (High)	0.630	0.210	3.00	0.003	1.88
Socioeconomic Status (Medium)	0.318	0.200	1.59	0.111	1.37
Parental Education (University)	0.527	0.185	2.85	0.004	1.69
Parental Education (Secondary)	0.189	0.180	1.05	0.294	1.21
Psychological Health (Good)	0.676	0.190	3.56	<0.001	1.97
Psychological Health (Moderate)	0.312	0.200	1.56	0.118	1.37
Urban Residence (Yes)	0.402	0.180	2.23	0.026	1.50
Log-likelihood	−298.12				
AIC (Akaike Information Criterion)	628.24				
Pseudo R^2^ (Nagelkerke)	0.36				
Sample Size (n)	636				
Significance Level	*p* < 0.05 (statistically significant)				

## Data Availability

The raw data supporting the conclusions of this article will be made available by the authors on request due to privacy.
